# The Experience of Frail Older Patients in the Boarding Area in the Emergency Department: A Qualitative Systematic Review

**DOI:** 10.3390/jcm14103556

**Published:** 2025-05-19

**Authors:** Pasquale Iozzo, Giovanna Cannizzaro, Stefano Bambi, Luana Maria Amato, Simona Fanuli, Dhurata Ivziku, Giuliano Anastasi, Alberto Lucchini, Noemi Spina, Roberto Latina

**Affiliations:** 1 Department of Biomedicine and Prevention, University of Rome “Tor Vergata”, Via Montpellier, 1, 00133 Rome, Italy; 2 Anesthesia and Intensive Care Unit, Emergency Department, Azienda Ospedaliera Universitaria Policlinico “Paolo Giaccone”, Via del Vespro, 129, 90127 Palermo, Italy; giovanna.cannizzaro@policlinico.pa.it (G.C.); noemi.spina@policlinico.pa.it (N.S.); 3 Department of Health Sciences, University of Florence, Viale GB Morgagni, 48, 50134 Florence, Italy; stefano.bambi@unifi.it; 4 Villa Sofia-Cervello United Hospitals, Viale Strasburgo, 233, 90127 Palermo, Italy; luanamaria.amato@community.unipa.it (L.M.A.); simona.fanuli@community.unipa.it (S.F.); 5 Department of Health Professions, Fondazione Policlinico Universitario Campus Bio-Medico, 00128 Rome, Italy; d.ivziku@policlinicocampus.it; 6 Department of Trauma, AOU G. Martino University Hospital, 98124 Messina, Italy; giuliano.anastasi.it@gmail.com; 7 Emergency and Urgency Department-Health Professions Department, A.O. San Gerardo, 20126 Monza, Italy; alberto.lucchini@unimib.it; 8 Department of Health Promotion Science, Maternal and Infant Care, Internal Medicine, and Medical Specialities (PROMISE), University of Palermo, Piazza delle Cliniche, 2, 90127 Palermo, Italy; roberto.latina@unipa.it

**Keywords:** frail older adult, emergency department boarding, patient experience, qualitative systematic review, JBI

## Abstract

**Background/Objectives:** Boarding refers to the period when patients deemed stable in the emergency department (ED) are temporarily monitored, wait to be admitted, and receive appropriate care. As life expectancy increases, so does the importance of understanding the dynamics and experiences of older adults with frailty in emergency settings. The absence of a care environment tailored to specific needs could diminish the overall quality of care provided, threatening the health and well-being of this population. To our knowledge, how frail older adults experience this has not yet been synthesized in a qualitative systematic review. The aim of this study was to explore the lived experiences of frail older adults during the emergency department (ED) boarding phase **Methods:** This systematic review was conducted using PubMed, OVID, and Scopus in October 2024. No time restrictions were settled and only articles published in English were included. Following the predefined inclusion criteria, two researchers independently extracted and synthesized the data using the Joanna Briggs Institute (JBI) meta-aggregation methodology and instruments. **Results:** Seven studies were included. Thirty-one findings were identified and grouped into seven categories and three themes regarding the lived experiences of frail people in ED boarding areas. The themes we identified were discomfort, distress, frustration, the experience of positive/negative attitudes of healthcare providers, and the supportive role of family members during ED LOS (length of stay). **Conclusions:** Older frail adults experience significant physical and psychological distress during ED boarding. It is necessary to manage their specific needs through targeted actions aimed at improving their overall experience and quality of care in emergency settings.

## 1. Introduction

Emergency departments (EDs) worldwide face significant challenges in providing timely and quality care. This is largely due to the increasing number of patients and limited hospital resources [1]. The increasing number of older adults coupled with more patients suffering from multiple chronic conditions is one of the main factors contributing to the increase in ED visits and subsequent overcrowding [2,3]. Research has shown that overcrowding can lead to delays in care, lower adherence to clinical guidelines, higher mortality rates, and worse overall health outcomes in frail older patients [1,3,4]. Implementing strategies to improve patient flow in EDs has been shown to improve the quality of care, particularly for older adults, who are more vulnerable to the negative effects of ED overcrowding [1,2]. It is estimated that by 2050, 22% of the world’s population will be aged 65 years or older, significantly increasing the demand for healthcare services, particularly emergency care [5]. What makes this scenario more complex is that globally, advances in healthcare and living conditions have resulted in an aging population with increasing levels of frailty. Currently, approximately one-quarter of all ED patients worldwide are older adults, with many multimorbidities and polypharmacy [6]. This makes the diagnosis, treatment, and management of frail older adults more challenging [6,7]. Additionally, older adults are more likely to present with high-acuity conditions and atypical symptoms, which increases both healthcare costs and the use of ED resources [8]. Nurses and healthcare professionals are concerned that EDs may not always be the most appropriate setting for frail older patients because the provided care may not meet their specific needs [9]. To meet this growing demand and reduce the risks that older adults face when seeking care, it is essential that ED environments and care processes are adapted to meet their specific needs [9,10]. Understanding the experiences of older patients in the emergency department is therefore crucial for improving the quality of care they receive. However, knowledge of this topic is limited [11]. While the existing literature highlights challenges related to ED overcrowding and care for older adults [6,8,9,10], there is limited focus on how frail individuals uniquely perceive and experience prolonged waits. Given the projected rise in older patients utilizing ED, this systematic review aims to synthesize qualitative findings that illuminate the key dimensions of boarding experiences for frail adults in ED settings. In doing so, it seeks to provide actionable insights that inform patient-centered, age-sensitive care practices in ED settings.

## 2. Materials and Methods

This systematic literature review was performed using the Preferred Reporting Items for Systematic Reviews and Meta-Analyses (PRISMA) guidelines [12]. The Joanna Briggs Institute (JBI) meta-aggregative methodology and a critical interpretive approach were used to extract and synthesize the qualitative evidence [13]. An initial search of the JBI Evidence Synthesis, Cochrane Database, CINAHL, PubMed, and PROSPERO databases was performed to identify any existing systematic reviews on the topic, and no similar reviews were identified.

## 3. Inclusion Criteria

### 3.1. Types of Participants

The identified population included frail individuals aged 60 years or older who were admitted to the ED and were boarding there while waiting for an inpatient bed. Although frailty is of growing importance in the scientific field, its definition and application remain heterogeneous. As frailty is neither systematically evaluated nor uniformly defined in the literature, it introduces potential interpretative bias and constitutes a methodological limitation for comparative analyses between studies. Nevertheless, all the articles included in this paper addressed the concept to some extent, either by providing an explicit definition or by enabling an operational definition to be extrapolated from the described criteria, thus facilitating consistent integration within the analysis. The assessment of frailty in EDs remains a subject of debate within the scientific community as no universal definition of frailty exists [4]. Therefore, the definition we considered from the scientific literature on frailty is a state of latent vulnerability that significantly increases the risk of falls, loss of autonomy, disability, and a higher likelihood of acute hospital admission and death [4,6,7,10,14].

### 3.2. Phenomenon of Interest

The phenomenon of interest we considered was the experience of frail older individuals boarding in the ED while awaiting admission. This review focuses specifically on the perspectives, emotions, and challenges faced by these patients, with the aim of understanding their unique needs during prolonged ED stays. Qualitative studies that explore general experiences related to the physical, psychological, and social aspects of boarding in the ED were considered relevant to capture a holistic understanding of the patient experience.

### 3.3. Context

The identified context was the boarding area of the ED. Boarding refers to holding patients in the ED or a designated area while awaiting an inpatient bed [15,16], while length of stay (LOS) indicates the total time spent in the ED from arrival to discharge. As highlighted in this review, many studies refer to boarding or LOS, without providing a precise definition. Therefore, both terms were considered. Despite being a crucial aspect of care pathway organization, the concept of boarding presents significant terminological and conceptual variability in the literature. The absence of a standardized definition and shared criteria for identification represents a significant methodological limitation, reducing the possibility of making homogeneous comparisons between the results of various studies. Despite this critical issue, all articles included in the analysis addressed the topic of boarding, either directly or indirectly. Some provide a formal definition, while others reconstruct its operational meaning based on the described characteristics and conditions. This enabled consistent integration during data processing.

### 3.4. Types of Studies

Qualitative studies such as phenomenology, descriptive qualitative research, grounded theory, ethnography, phenomenography, and action research were considered. For mixed-methods studies, only qualitative results were included. No time restrictions were applied and only articles published in English were considered. There were no geographic limitations on the included studies. In contrast, incomplete studies and those not available in the full text were excluded. No studies reporting the opinions of healthcare providers or caregivers were included, as the primary interest was the firsthand experiences of older patients themselves. Moreover, theses, dissertations, abstracts in proceedings, unpublished papers, narrative reviews, editorials, letters to the editor, and commentaries were excluded.

## 4. Search Strategy and Study Selection

**Figure 1:**
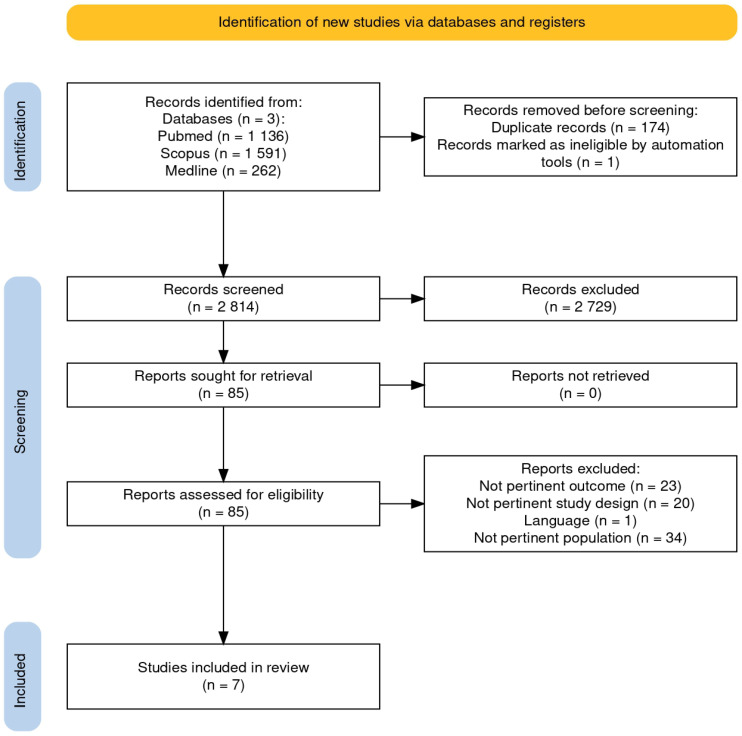
PRISMA flow diagram.

In this review, only English-language articles were included, and the search was conducted until October 2024 without applying any time filters. The following three databases were screened: PubMed, OVID, and Scopus. The search strategy for each database followed the same set of keywords adapted to the syntax rules of each platform. The complete search strategy is detailed in Appendix A. The article selection process was performed according to the PRISMA Statement. All citations were imported into Mendeley Desktop and the Rayyan web application [17] to facilitate independent screening by reviewers, and duplicates were removed. Title and abstract screening was performed by two independent reviewers (G.C. and N.S.), who assessed all studies based on the inclusion and exclusion criteria, excluding those that did not meet the criteria. Each study was deemed relevant based on its title or abstract and was further evaluated by two independent reviewers (N. S. and G. C.) to assess their eligibility. Full-text articles that did not meet the inclusion criteria were excluded, and the rationale for exclusion is provided in the PRISMA flow diagram (Figure 1). Any disagreements between reviewers were resolved through discussion. The authors of these studies were not contacted for additional information.

### 4.1. Assessment of Methodological Quality

All studies included in the review were evaluated for methodological quality using the JBI Qualitative Appraisal Tool for Qualitative Research [13]. This tool evaluates the studies against 10 quality criteria with a scoring option such as “yes”, “no”, unclear, or “not applicable”. Two reviewers (G.C. and P.I.) independently conducted these evaluations. Any discrepancies between reviewers were resolved through a consensus meeting. No study was excluded based on the analysis. The results of the critical appraisal of the studies are presented in Supplementary Materials.

### 4.2. Data Extraction

An electronic data extraction form, prepared and agreed upon by the authors prior to the systematic review, was used for general details of the study information extraction. Two independent reviewers (N.S. and G.C.) extracted the following information from each included study: author name, year of publication, country, study design, sample and setting, and number of participants. This information is summarized in Table 1. Subsequently, the two reviewers independently read the results of the included studies multiple times to extract the findings. According to the JBI methodology, a finding refers to a verbatim extract that captures the author’s analytical interpretation of the data. Each extracted finding from a study is paired with an accompanying illustration regarding a direct quotation from a participant, fieldwork observation, or other supporting evidence presented in the original study. Each extracted finding is evaluated for credibility and can be classified as follows: unequivocal (U)—evidence beyond reasonable doubt, encompassing findings that are factual, directly reported, or observed and not open to dispute; credible (C)—findings that are plausible and logically derived from the data and theoretical framework but remain open to challenge due to their interpretive nature; not supported (NS)—applies when neither of the above criteria are met, particularly when findings lack support from the data. These findings cannot be included in the meta-aggregative process according to the JBI.

### 4.3. Data Synthesis

Two reviewers independently assembled the identified findings and clustered them into categories based on the similarities in their meanings or concepts. Each category was accompanied by an explanatory statement that captured the comprehensive and inclusive meaning of the grouped findings. Similar categories were grouped in a synthesized finding accompanied by an explanatory statement that encapsulated the overall inclusive meaning. This meta-aggregative framework accurately reflects the aggregation of data from primary studies. At the end of the process, the reviewers compared the generated categories and, in case of discrepancies, held a discussion with a third researcher to reach an agreement.

## 5. Results

### 5.1. Search Results

The search identified 2989 records, of which 174 were duplicates. Ultimately, seven studies were included in this systematic review, all of which were published between 2004 and 2023. The included studies consist of five qualitative studies, one pilot field study, and one mixed-methods study. One study originated in Ireland, and the remaining six originated in the UK, Canada, Ghana, Sweden, the USA, and the Netherlands. The PRISMA flow diagram [24] in Figure 1 outlines the search results as well as the process of study selection and inclusion. The studies included a sample of 1224 frail older people with a total of 491 older patient experiences included in the systematic review. Most studies have used a qualitative descriptive approach. Further details are provided in Table 1. All the included studies received a high score on quality (8–9/10) with some levels of uncertainty referring to the cultural and theoretical location of the researchers or the influence of the researchers on the conducted research. See Supplementary Table S1 for further information.

### 5.2. Meta-Aggregation

The literature on the experiences and needs of older adults in EDs highlights several common themes and offers suggestions for improving the quality of care. This systematic review identified 31 findings that were grouped into seven categories and three synthesized findings (themes). Table 2 summarizes the process. Additionally, Table 2 describes the evaluation of the credibility of the findings and the synthesis for each finding included in the study. None of the findings were considered unsupported. Therefore, all the findings were included in the meta-analysis.

### 5.3. THEME 1: Waiting Time Among Frail Older Patients Typically Generates Discomfort, Distress, and Frustration

This synthesized theme included 11 findings and three categories of experiences connected to boarding waiting times.

#### 5.3.1. Discomfort

One of the most discussed aspects is discomfort caused by long waiting times and inadequate environmental conditions. Emergency departments are now characterized by overcrowding. Waiting time in the boarding area is categorized by a lack of privacy, with patients sitting in a chair or lying on a bed, in a waiting room or hallway, which prolonged over time, causing negative experiences for frail older patients [21]. In addition, the proximity of other sick patients is a cause of anxiety and discomfort [19]. Older adults often report that waiting in the ED is characterized by uncertainty and disorientation, and many experience this phase as an uncomfortable physical condition in crowded, noisy environments [22].

“The seats ... oh the seats were dreadful! Someone was lying on the floor in preference to sitting on the chairs because they were in such a lot of pain” [19].

#### 5.3.2. Distress

Waiting time in the boarding area further compromises frail older patients’ health and causes psychological distress [22]. This feeling of distress is accentuated by the long waiting time [8]. For example, a study conducted in Ireland found that waiting time was one of the main sources of dissatisfaction among older patients, especially when they did not receive adequate information regarding their treatment status [22].

“Just the thought of having to wait in the corridor ... just waiting there. No, I did not like that. Because everybody’s walking by you and they’re looking at you as if to say, What’s wrong with her?” [19].

“You feel deserted, you just sit there, and nothing happens” [8].

#### 5.3.3. Frustration

Older patients reported that their need to be seen increased because they were not always informed of the reasons for long waiting times, leading to a sense of frustration. Older patients showed a great desire for contact with staff, sought out to make eye contact with nurses, frequently looked at doors, or listened to someone’s footsteps [21]. The feeling of frustration is also caused by not having a sense of time, not having personal belongings or clothing with them, being asked many questions, and going to multiple healthcare providers [8].

“Maybe I let myself be taken by surprise again by being in the hospital, like a pathetic bunch of human beings” [8].

“...to sit here and wait, and the only contact I have with the staff is when they carry out tests on me, you feel that you’re not being seen as a person...” [21].

### 5.4. THEME 2: Experience with Positive and Negative Attitudes of Healthcare Providers

The attitudes of healthcare staff can significantly influence the overall experience of older adults. The experience was evidenced by 13 findings that were grouped into two categories.

#### 5.4.1. Lack of Empathy Among Health Professionals

Several studies have reported that many older patients feel disrespected, ignored, and stigmatized [18,19,23]. For example, in Ghana, many older patients perceive healthcare staff as lacking compassion and showing little patience and attention to them [20]. This negatively affects their overall experience and contributes to the perceived sense of invisibility among these patients [8].

“Increase in staff numbers is required urgently. Patients cannot receive appropriate care when staff are so busy dealing with the large number of patients presenting in ED” [20].

#### 5.4.2. Lack of Communication

Communication between patients and healthcare professionals is a critical issue. Many older adults reported a lack of clarity and transparency regarding their care. Studies in different contexts have shown that patients want to be constantly informed about their health status and waiting time [8,18,19,20].

“For doctors and nurses, it is appropriate that when an older person visits a hospital, the nurses reckon that this is an older person; they cannot put you among young people. They should give you extra attention, communication, treat you well, and give you good medical care so you can return home and rest” [20].

However, not all feedback is negative; some studies report that when care is personalized and healthcare professionals take the time to explain conditions and treatments, older patients’ experiences are positive. In a study conducted in a large US hospital, the introduction of interventions such as bedside care coordination led to significant improvements in patient satisfaction [25]. One study examined the effectiveness of early intervention by an interdisciplinary team of health and social care professionals and found significant improvements in ED length of stay and reductions in hospital admission rates. These interventions also improved the mobility and autonomy of older adults in the weeks following the ED visit, demonstrating the importance of an integrated approach to care [26].

### 5.5. THEME 3: Supportive Role of Family Members During ED LOS

Another important aspect of boarding frail older adults is the involvement of their family members. Two categories and seven findings were grouped into this synthesized finding.

#### 5.5.1. Involvement of Family Members

Acquiring and understanding patient and family perspectives and achieving a shared understanding of the problem results in improving the relationship with healthcare providers [18]. Older adults and their caregivers emphasized the importance of being involved in the decision-making process. Studies suggest that active involvement of family members in decision-making improves quality of care, especially at discharge, and ensures a safer transition from hospital to home [19].

“If my family members wanted to talk to a doctor, they had difficulty doing so” [18].

#### 5.5.2. Frail Older Patient Experience of Boarding Improved by Family Members

Frail older patients waiting in the ED need assistance with basic needs, such as eating or going to the bathroom. The presence of a family member allows these patients to perform these activities [22]. Moreover, Cassarino et al. [25] found that reasons for ED attendance among “lower urgency” older patients are warranted by lower levels of social and family support in the community.

“It is nice to have someone with you. They can help me remember things or correct me if I downplay the situation. My son always does this. Two can remember more than one, and he asks questions that I do not think of. I find that helpful” [8].

## 6. Discussion

This qualitative systematic review examined the lived experiences of older frail patients in the boarding area of the emergency department. The characteristics of adults who frequently attend EDs have been described in several studies and can be traced to patients aged ≥ 60 years. This is because the population growth of older patients characterizes the population of many countries [26]. The meta-aggregation of findings identified three areas of experience: waiting time, positive and negative attitudes of healthcare providers, and the supportive role of family members during ED LOS. A common challenge faced by frail older people in the boarding areas of EDs is the long waiting time. The length of stay (LOS) in the emergency room has a negative impact on the satisfaction with care experienced by older patients and is particularly sensitive to examination waiting times, which are strongly influenced by overcrowded services [23]. In a mixed-method study [22], the qualitative component showed that older patients expressed concern about poor-quality ED services and emphasized the need for immediate improvement in waiting time, personal care, and privacy. Moreover, if the waiting period was longer than six hours, older patients were significantly more likely to report poor ED experiences. McCusker et al., 2018 [18], in their qualitative study, showed that informing the older patient and family about waiting times can reduce their expectations and avoid the phenomenon of dissatisfaction. At times, frail older patients found the experience of waiting particularly psychologically taxing, adding to the state of physical discomfort involving the emotional sphere. Many older patients reported that their basic needs were not met and that they did not receive basic nursing care such as nutrition, pain relief, empathy, interest in their living situations, and information on self-care. For patients who are not classified as immediate and must wait several hours, it may be important for their well-being to be able to obtain food and drink while boarding [27]. This is the first meta-synthesis to highlight waiting time as the primary cause of dissatisfaction. In a qualitative study by Issahaku et al. [20], participants argued that their advanced age makes them weak and vulnerable; therefore, they should not be forced into long waits for service nor shuffled into queues with younger people. The participants called for better treatment from healthcare providers based on an age-friendly approach by creating dedicated hospital pathways.

In addition, older patients described the positive and negative attitudes of healthcare providers. The findings of Kihlgren et al. [21] illustrate the importance of nursing care and how it is provided. Patient satisfaction can be improved through nursing actions that include empathy and the ability to understand a patient’s needs. Healthcare providers must demonstrate situational awareness to care for frail older patients, without patients explicitly asking for it. To do so, they need to understand two priority aspects: how life experiences influence the values of older patients and how they want to be treated, investigate what is needed to meet this need for situational awareness, and the influence on shared decision-making and patient-centered care in the emergency department [8]. In the study by Issahaku et al. [20], participants (adults aged 60 years and over) were asked to suggest ways in which healthcare professionals could improve their delivery of care to frail older adults. The need for compassionate treatment was emphasized among their priorities. Compassionate care is described as respect, honesty, and patience. One patient specifically requested that healthcare professionals be honest and transparent about the prognosis of their condition. Additionally, frail older patients were considered to require priority attention because of their vulnerable health status and the urgency of returning home as soon as possible. Some participants also suggested developing dedicated care pathways, specifically for frail older individuals. One proposed solution to enhance the quality of care in emergency departments was the establishment of “geriatric emergency departments” or “older patients’ emergency rooms” for individuals over the age of sixty-five. However, this is only one of several possible approaches. Another important strategy is to strengthen the geriatric competencies of existing emergency care staff through targeted training to ensure that frailty is recognized and managed properly within standard emergency settings. A multifaceted approach that combines environmental adaptations and workforce development may be the most effective way forward.

Emergency department planning focuses on rapid patient assessment, stabilization, and eventual admission or discharge. Patient privacy and comfort are abandoned owing to greater maneuverability and staff flexibility [28]. Geriatrizing the traditional emergency department, with interdisciplinary training for the diseases that most commonly characterize older patients, such as cognitive decline, with structural changes, facilitating their access, and resulting in a familiar environment, has been shown to improve the quality of care and safety of older adults while reducing waiting time and costs [28]. The availability of a geriatric specialist team, emergency geriatric, or emergency service can help avoid unnecessary transfers that may result in potentially avoidable transfer-related adverse events. Until such services are available in our healthcare system, it will not be possible to deliver care appropriate to the chronic conditions of frail older patients [29]. The need for families of frail older patients waiting in the emergency room is an essential issue. In fact, McCusker et al. investigated this aspect [18]. The number of patients who required the presence of a family member at the bedside increased as waiting time increased.

From the study, it is shown that this aspect should be explored in detail because it is important to prevent the deterioration of physical and mental health during ED stay to prevent post-ED functional decline. Misinformation seems to play a crucial role in older adults’ dissatisfaction during their stay in the ED. This is confounded and amplified by cognitive dysfunction, geriatric physical demands, and lower health literacy. Eliminating barriers to communication, waiting time, communication content, and patient education seems to have a positive impact on patient experience, and this is possible through the involvement of caregivers [30]. An older patient-friendly health service will require age awareness and geriatric training for physicians and nurses, covering several levels, with a priority healthcare policy for older and frail adults waiting in the emergency department.

## 7. Limitations

Despite the importance of this systematic review, this study had some limitations. This systematic review included only studies published in English, which may indicate that potentially relevant studies conducted in non-English-speaking countries may have been overlooked. Additionally, the research was conducted using only three databases; therefore, some qualitative studies published in other databases may have been missed. Furthermore, studies have reported that it is difficult to recruit acutely ill older patients in a fast-paced emergency environment; therefore, the study sample analyzed was limited. In addition, the boarding experience of older adults is inevitably influenced by the structural organization of emergency departments and the broader healthcare system within each country. Variations in access to geriatric care pathways, the availability of inpatient beds, staffing models, and cultural attitudes toward aging may affect both the nature and outcome of ED boarding. Consequently, while our findings highlight several common themes, caution should be exercised when generalizing the results across different healthcare settings. Future research should consider cross-national comparisons to better understand the context-specific factors influencing the boarding experience. Furthermore, future studies should consider including research from non-English-speaking countries and expanding the range of databases searched to provide a more comprehensive review. Many studies have used small sample sizes, limiting the robustness of their findings. Cultural and contextual differences across the study settings may also influence the applicability of the results to different populations. Furthermore, methodological heterogeneity, including variations in the definition and measurement of frailty and boarding, introduces potential bias. These factors should be considered when interpreting synthesized experiences and drawing conclusions.

## 8. Implication for Clinical Practice

Our findings underline the urgent need to translate research into real-world practices that better support frail older adults during ED boarding. Simple but targeted interventions—such as early geriatric assessments, age-friendly environmental adjustments, clear communication, geriatric care pathways, and greater family involvement—can significantly improve patient experiences and outcomes. Integrating interdisciplinary teams and promoting staff training in geriatric care are practical and scalable strategies that can be adapted to different healthcare systems. Bridging the gap between evidence and everyday clinical practice is essential to make emergency departments safer and more responsive to the aging population. Additionally, future research examining the specific barriers and challenges faced in recruiting older patients in various healthcare settings could also provide valuable insights. Moreover, exploring the perspectives of healthcare providers and patients on age-friendly practices could help to refine and improve the implementation of such approaches in clinical settings.

## 9. Conclusions

In conclusion, a holistic approach that considers the physical, psychological, and social needs of frail older people in the ED boarding area is fundamental to improving their care. A more comfortable environment, clear and transparent communication, and greater involvement of family members could ensure a positive experience and improve quality of care. Solutions involving interdisciplinary teams and individualized measures could significantly improve the experience of older adults in the ED and reduce the risks associated with their hospital stay. Future studies should investigate the long-term effects of these holistic approaches on patient outcomes and satisfaction. Additionally, research could focus on identifying the specific barriers to implementing such approaches in different healthcare settings and developing strategies to overcome these challenges. Comparative studies on different EDs that have adopted age-friendly practices could provide valuable insights into the effectiveness of these measures. Exploring the perspectives of healthcare providers, older patients, and their families regarding the proposed solutions could further inform and refine these approaches.

## Figures and Tables

**Table 1:** Summary of the studies included in this review.

Author (Year), Country	Aim	Design	Sample and Setting	Methods	Experience Main Themes
McCusker et al. (2018), [18] Canada	Evaluate older adults’ experiences of problems in the ED.	Qualitative study (focus group)	≥75 years, ED *N* = 412 F = 275	The authors used a 16-question questionnaire to assess the two dimensions (personal care/communication and waiting time).	Areas of experience that are more relevant to older people are family needs (e.g., keeping informed, participating in care), physical needs (e.g., thirst, mobilization, physical environment) and transitional care needs (e.g., patient-centerd discharge information, communication with doctor).
Graham et al. (2023) [19], England	The study evaluates the experience of older adult patients in the emergency department.	Qualitative study (interviews)	≥65 years, ED *N* = 24 F = 15	Using semi-structured interviews with three main questions, assessing three dimensions: patient experience, patient safety, and clinical effectiveness.	Questions exploring patient experiences of care confirmed that meeting the communication, care, waiting, physical, and environmental needs were prominent determinants of experience for older adults.
Issahaku& Sulemana (2021) [20], Ghana	The study evaluates the experience of older adult patients in the emergency department	Qualitative study (interviews)	≥60 years, ED *N* = 23 F = 9	Using semi-structured interviews with two main questions. The experiences of hospital visits and how they would like to be treated by health professionals are the focus of this paper.	Uncompassionate care, disrespectful attitudes, and a better way of treating us emerged as three main themes from the data.
Kihlgren et al. (2004) [21], Sweden	The focus of the observations was on the patients, the care they received, the family members accompanying them, and the general environment.	Pilot field study (observations)	≥75 years, ED *N* = 20 F = 14	Open, non-participant observations were conducted by following the patient from the reception area to the examination room until discharge from the ED. Four researchers each conducted one observation in the ED for the pilot study.	Selective coding resulted in six core variables that became the focus of the analysis. These variables were: uncomfortable waiting, superfluous waiting, lack of good routines while waiting, suffering while waiting, negative emotions while waiting, and care while waiting.
Venema et al. (2023) [8], Netherlands	The study evaluates the experience of older adult patients in the emergency department	Qualitative study (interviews)	≥70 years, ED *N* = 12 F = 4	A qualitative design was used, and semi-structured interviews were conducted with discharged twelve frail older adult patients who had been admitted to the emergency department.	The themes of feeling disturbed, expecting care, suppressing needs and wanting to be seen emerged from the analysis. These themes suggested a need for healthcare professionals to be more aware of the situation when caring for participants. This was influenced by the participants’ life experiences.
Mwakilasa et al. (2021) [22], Ireland	The study evaluates the experience of older adult patients in the emergency department	Mixed methods study (qualitative interview component used)	≥ 65 years ED *N* = NR	This concurrent mixed-methods study involved secondary analysis of qualitative and quantitative data.	Scores for overall ED experiences were calculated from five questions that asked patients about communication, privacy, waiting time, and whether they were treated with dignity and respect in the ED.
Puppala et al. (2020) [23], USA	Care’s satisfaction of frail older adults.	Qualitative study (interview Survey)	≥65 years ED *N* = NR	A qualitative study with the inpatient population of an eight-hospital tertiary medical center in 2015. The satisfaction determinants were based on the Hospital Consumer Assessment of Healthcare Providers and Systems (HCAHPS) survey answers and included clinical and organizational variables.	The older groups with the lowest satisfaction score were those admitted through the ED who experienced long waiting times or had chronic diseases.

F = female. *N* = number of patients in the emergency department (ED). NR = not reported. HCAHPS = Hospital Consumer Assessment of Healthcare Providers and Systems.

**Table 2:** Description of themes, codes, and review findings.

Synthetized Theme	Categories	Findings
Waiting time experience among frail older patients	Discomfort	Lack of privacy [D1] U; Sitting in a chair or lying on a bed [B1] U [D2] U; Stay near sick patient [B2]C; Crowded environment [F1] U [D3]C
	Distressing	Long waiting time [E1] C [G1] C [F2] C [B3] U [D4] U [H1] U; Any information about treatment [F3]C; Psychological distress [F4]C
	Frustration	Do not know long waiting time’s reason [D5] C; Not have sense of time [D6] C; Not having personal belongings [H2] U; Physical needs [H3] U
Experience with attitude of healthcare providers	Lack of empathy among health professionals	Lacking compassion [C1] C; Trustworthiness [C2] C; Disrespectful Attitude [C3] C; Perceived sense of invisibility [A1] C; Showed a great longing for contact with staff [D7] C; Staff do not have enough time to talk to patients [A2] C
	Lack of communication	Lack of clarity and transparency about their care [B4] C; Wanting to be seen [H3] C; The level of patient satisfaction heavily depends on the adequacy of communication [G2] C; Inadequate communication [F5] U; Communication on health problem [E1] C; Use discriminatory communication [C4] C
The role of family members during ED LOS	Involvement of family members	The importance of being involved in the decision-making process [B5] U; Understanding patient and family perspectives [E2] U
	Frail older patient experience of boarding improved by family members	Lower level of social and family support [A3] C; Need assistance in basic needs [D8] U; Reduces sense of confusion [G3] U; They do not suppress their needs [H4] C

The capital letter, near the name of the authors of the articles, indicates the order in which the findings were presented in the original articles. U refers to unequivocal findings and C refers to credible findings. Cassarino et al. [A]; Graham et al. [B]; Issahaku et al. [C]; Kihlgren et al. [D]; McCusker et al. [E]; Mwakilasa [F]; Puppala et al. [G]; Venema et al. [H] [8,18,19,20,21,22,23,25].

## Data Availability

Not applicable.

## Supplementary Materials

The following supporting information can be downloaded at: https://www.mdpi.com/article/10.3390/jcm14103556/s1. Table S1: Appraisal results for the included studies using the JBI Critical Appraisal Checklist for Qualitative Research [13].

## Appendix A. Search Strategy Until 31 October 2024

**PUBMED** **MEDLINE** **SCOPUS**
(boarding OR length of stay) AND (emergency department OR emergency room OR emergency service) AND (elderly OR older adults) AND (experience OR patient experience OR patient satisfaction OR psychological impact OR patient perceptions) (“boarded”[All Fields] OR “boarding”[All Fields] OR (“length of stay”[MeSH Terms] OR (“length”[All Fields] AND “stay”[All Fields]) OR “length of stay”[All Fields])) AND (“emergency service, hospital”[MeSH Terms] OR (“emergency”[All Fields] AND “service”[All Fields] AND “hospital”[All Fields]) OR “hospital emergency service”[All Fields] OR (“emergency”[All Fields] AND “department”[All Fields]) OR “emergency department”[All Fields] OR(“emergency service, hospital”[MeSH Terms] OR (“emergency”[All Fields] AND “service”[All Fields] AND “hospital”[All Fields]) OR “hospital emergency service”[All Fields] OR (“emergency”[All Fields] AND “room”[All Fields]) OR “emergency room”[All Fields]) OR (“emergency medical services”[MeSH Terms] OR (“emergency”[All Fields] AND “medical”[All Fields] AND “services”[All Fields]) OR “emergency medical services”[All Fields] OR (“emergency”[All Fields] AND “service”[All Fields]) OR “emergency service”[All Fields])) AND (“aged”[MeSH Terms] OR “aged”[All Fields] OR “elderly”[All Fields] OR “elderlies”[All Fields] OR “elderly s”[All Fields] OR “elderlys”[All Fields] OR (“aged”[MeSH Terms] OR “aged”[All Fields] OR (“older”[All Fields] AND “adults”[All Fields]) OR “older adults”[All Fields])) AND (“experience”[All Fields] OR “experience s”[All Fields] OR “experiences”[All Fields] OR ((“patient s”[All Fields] OR “patients”[MeSH Terms] OR “patients”[All Fields] OR “patient”[All Fields] OR “patients s”[All Fields]) AND (“experience”[All Fields] OR “experience s”[All Fields] OR “experiences”[All Fields])) OR (“patient satisfaction”[MeSH Terms] OR (“patient”[All Fields] AND “satisfaction”[All Fields]) OR “patient satisfaction”[All Fields]) OR ((“psychologic”[All Fields] OR “psychological”[All Fields] OR “psychologically”[All Fields] OR “psychologization”[All Fields] OR “psychologized”[All Fields] OR “psychologizing”[All Fields]) AND (“impact”[All Fields] OR “impactful”[All Fields] OR “impacting”[All Fields] OR “impacts”[All Fields] OR “tooth, impacted”[MeSH Terms] OR (“tooth”[All Fields] AND “impacted”[All Fields]) OR “impacted tooth”[All Fields] OR “impacted”[All Fields])) OR ((“patient s”[All Fields] OR “patients”[MeSH Terms] OR “patients”[All Fields] OR “patient”[All Fields] OR “patients s”[All Fields]) AND (“percept”[All Fields] OR “perceptibility”[All Fields] OR “perceptible”[All Fields] OR “perception”[MeSH Terms] OR “perception”[All Fields] OR “perceptions”[All Fields] OR “perceptional”[All Fields] OR “perceptive”[All Fields] OR “perceptiveness”[All Fields] OR “percepts”[All Fields])))

## References

[1] Burgess L., Ray-Barruel G., Kynoch K. (2022). Association between emergency department length of stay and patient outcomes: A systematic review. Res. Nurs. Health.

[2] Nouri Y., Gholipour C., Aghazadeh J., Khanahmadi S., Beygzadeh T., Nouri D., Nahaei M., Karimi R., Hosseinalipour E. (2020). Evaluation of the risk factors associated with emergency department boarding: A retrospective cross-sectional study. Chin. J. Traumatol. Engl. Ed..

[3] Ruffo R.C., Shufflebarger E.F., Booth J.S., Walter L.A. (2022). Race and Other Disparate Demographic Variables Identified Among Emergency Department Boarders. West. J. Emerg. Med..

[4] Iozzo P., Spina N., Cannizzaro G., Gambino V., Patinella A., Bambi S., Vellone E., Alvaro R., Latina R. (2024). Association between Boarding of Frail Individuals in the Emergency Department and Mortality: A Systematic Review. J. Clin. Med..

[5] Liu H., Shang N., Chhetri J.K., Liu L., Guo W., Li P., Guo S., Ma L. (2020). A Frailty Screening Questionnaire (FSQ) to Rapidly Predict Negative Health Outcomes of Older Adults in Emergency Care Settings. J. Nutr. Health Aging.

[6] Cardona M., Lewis E.T., Kristensen M.R., Skjøt-Arkil H., Ekmann A.A., Nygaard H.H., Jensen J.J., Jensen R.O., Pedersen J.L., Turner R.M. (2018). Predictive validity of the CriSTAL tool for short-term mortality in older people presenting at Emergency Departments: A prospective study. Eur. Geriatr. Med..

[7] van Dam C.S., Trappenburg M.C., ter Wee M.M., Hoogendijk E.O., de Vet H.C., Smulders Y.M., Nanayakkara P.W., Muller M., Peters M.J. (2021). The Accuracy of Four Frequently Used Frailty Instruments for the Prediction of Adverse Health Outcomes Among Older Adults at Two Dutch Emergency Departments: Findings of the AmsterGEM Study. Ann. Emerg. Med..

[8] Venema D., Vervoort S.C.J.M., de Man-van Ginkel J.M., Bleijenberg N., Schoonhoven L., Ham W.H.W. (2023). What are the needs of frail older patients in the emergency department? A qualitative study. Int. Emerg. Nurs..

[9] García-Peña C., Pérez-Zepeda M.U., Robles-Jiménez L.V., Sánchez-García S., Ramírez-Aldana R., Tella-Vega P. (2018). Mortality and associated risk factors for older adults admitted to the emergency department: A hospital cohort. BMC Geriatr..

[10] Maarek L., Maillet F., Turki A., Altar A., Hamdi H., Berroukeche M., Haguenauer D., Chemouny M., Cailleaux P.-E., Javaud N. (2020). Prognosis of non-severely comorbid elderly patients admitted to emergency departments: A prospective study. Am. J. Emerg. Med..

[11] Lilleheie I., Debesay J., Bye A., Bergland A. (2020). A qualitative study of old patients’ experiences of the quality of the health services in hospital and 30 days after hospitalization. BMC Health Serv. Res..

[12] Page M.J., McKenzie J.E., Bossuyt P.M., Boutron I., Hoffmann T.C., Mulrow C.D., Shamseer L., Tetzlaff J.M., Akl E.A., Brennan S.E. (2021). The PRISMA 2020 statement: An updated guideline for reporting systematic reviews. BMJ.

[13] Aromataris E., Lockwood C., Porritt K., Pilla B., Jordan Z.E. (2024). JBI Manual for Evidence Synthesis. JBI. https://synthesismanual.jbi.global.

[14] Lewis S., Evans L., Rainer T., Hewitt J. (2020). Screening for frailty in older emergency patients and association with outcome. Geriatrics.

[15] Janke A.T., Melnick E.R., Venkatesh A.K. (2022). Hospital Occupancy and Emergency Department Boarding during the COVID-19 Pandemic. JAMA Netw. Open.

[16] Joseph J.W., Rosen A., Kennedy M. (2025). Boarding in the Emergency Department: Specific Harms to Older Adults and Strategies for Risk Mitigation. Emerg. Med. Clin. N. Am..

[17] Ouzzani M., Hammady H., Fedorowicz Z., Elmagarmid A. (2016). Rayyan—A web and mobile app for systematic reviews. Syst. Rev..

[18] McCusker J., Cetin-Sahin D., Cossette S., Ducharme F., Vadeboncoeur A., Vu T.T.M., Veillette N., Ciampi A., Belzile E., Berthelot S. (2018). How Older Adults Experience an Emergency Department Visit: Development and Validation of Measures. Ann. Emerg. Med..

[19] Graham B., Smith J.E., Nelmes P., Squire R., Latour J.M. (2023). Initial Development of a Patient Reported Experience Measure for Older Adults Attending the Emergency Department: Part I—Interviews with Service Users. Healthcare.

[20] Issahaku P.A., Sulemana A. (2021). Older Adults’ Expectations and Experiences With Health care Professionals in Ghana. SAGE Open.

[21] Kihlgren A.L., Nilsson M., Skovdahl K., Palmblad B., Wimo A. (2004). Older patients awaiting emergency department treatment. Scand. J. Caring Sci..

[22] Mwakilasa M.T., Foley C., O’carroll T., Flynn R., Rohde D. (2021). Care Experiences of Older People in the Emergency Department: A Concurrent Mixed-Methods Study. J. Patient Exp..

[23] Puppala M., Ezeana C.F., Alvarado M.V.Y., Goode K.N., Danforth R.L., Wong S.S., Vassallo M.L., Wong S.T. (2020). A multifaceted study of hospital variables and interventions to improve inpatient satisfaction in a multi-hospital system. Medicine.

[24] Haddaway N.R., Page M.J., Pritchard C.C., McGuinness L.A. (2022). PRISMA2020: An R package and Shiny app for producing PRISMA 2020-compliant flow diagrams, with interactivity for optimised digital transparency and Open Synthesis. Campbell Syst. Rev..

[25] Cassarino M., Robinson K., Trépel D., O’shaughnessy Í., Smalle E., White S., Devlin C., Quinn R., Boland F., Ward M.E. (2021). Impact of assessment and intervention by a health and social care professional team in the emergency department on the quality, safety, and clinical effectiveness of care for older adults: A randomised controlled trial. PLoS Med..

[26] Street M., Berry D., Considine J. (2018). Frequent Use of Emergency Departments by Older People: A Comparative Cohort Study of Characteristics and Outcomes. Int. J. Qual. Heal. Care.

[27] Muntlin Å., Gunningberg L., Carlsson M. (2006). Patients’ Perceptions of Quality of Care at an Emergency Department and Identification of Areas for Quality Improvement. J. Clin. Nurs..

[28] Hwang U., Shah M.N., Han J.H., Carpenter C.R., Siu A.L., Adams J.G. (2013). Transforming Emergency Care for Older Adults. Health Aff..

[29] Ukkonen M., Jämsen E., Zeitlin R., Pauniaho S.L. (2019). Emergency Department Visits in Older Patients: A Population-Based Survey. BMC Emerg. Med..

[30] Berning M.J., e Silva L.O.J., Suarez N.E., Walker L.E., Erwin P., Carpenter C.R., Bellolio F. (2020). Interventions to Improve Older Adults’ Emergency Department Patient Experience: A Systematic Review. Am. J. Emerg. Med..

