# Supplementary material for: The Experience of Frail Older Patients in the Boarding Area in the Emergency Department: A Qualitative Systematic Review
Source: J Clin Med. 2025 May 19;14(10):3556. doi: 10.3390/jcm14103556 (PMC12112660; doi:10.3390/jcm14103556)
Supplement: Supplementary file 1 [file jcm-14-03556-s001.zip › jcm-3584448-supplementary.pdf]

Table S1. Appraisal results for the included studies using the JBI Critical Appraisal Checklist for Qualitative Research (Aromataris E, Lockwood C, Porritt K, Pilla B, Jordan Z, 2024).

Study	*Q1*	*Q2*	*Q3*	*Q4*	*Q5*	*Q6*	*Q7*	*Q8*	*Q9*	*Q10*	*Overall*
Graham et al. 2023 [19]	***Y***	***Y***	***Y***	***Y***	***Y***	***U***	***U***	***Y***	***Y***	***Y***	***8/10***
Issahaku & Suleman 2021 [20]	***Y***	***Y***	***Y***	***Y***	***Y***	***Y***	***U***	***Y***	***Y***	***Y***	***9/10***
Kihlgren et al. 2004 [21]	***Y***	***Y***	***Y***	***Y***	***Y***	***U***	***U***	***Y***	***Y***	***Y***	***8/10***
McCusker et al. 2018 [18]	***Y***	***Y***	***Y***	***Y***	***Y***	***U***	***Y***	***Y***	***Y***	***Y***	***9/10***
Mwakilasa et al. 2021 [19]	***Y***	***Y***	***Y***	***Y***	***Y***	***U***	***U***	***Y***	***Y***	***Y***	***8/10***
Puppala et al. 2020 [23]	***Y***	***Y***	***Y***	***Y***	***Y***	***Y***	***U***	***Y***	***Y***	***Y***	***9/10***
Venema et al. 2023 [8]	***Y***	***Y***	***Y***	***Y***	***Y***	***U***	***U***	***Y***	***Y***	***Y***	***8/10***

LEGEND: Y=yes, N=no, U=Unclear, NA=Not Applicable. Q1. Is there congruity between the stated philosophical perspective and the research methodology? Q2. Is there congruity between the research methodology and the research question or objectives? Q3. Is there congruity between the research methodology and the methods used to collect data? Q4. Is there congruity between the research methodology and representation and analysis of data? Q5. Is there congruity between the research methodology and the interpretation of the results? Q6. Is there a statement locating the researcher culturally or theoretically? Q7. Is the influence of the researcher on the research and vice-versa addressed? Q8. Are participants and their voices adequately represented? Q9. Is the research ethical according to current criteria or for recent studies, and is there evidence of ethical approval by an appropriate body? Q10. Do the conclusions drawn in the research report flow from the analysis or interpretation of the data?
